# The positional identity of mouse ES cell-generated neurons is affected by BMP signaling

**DOI:** 10.1007/s00018-012-1182-3

**Published:** 2012-10-16

**Authors:** Michele Bertacchi, Luca Pandolfini, Elisa Murenu, Alessandro Viegi, Simona Capsoni, Alessandro Cellerino, Andrea Messina, Simona Casarosa, Federico Cremisi

**Affiliations:** 1 Scuola Normale Superiore di Pisa, Piazza dei Cavalieri 7, 56100 Pisa, Italy; 2 CIBIO, Università di Trento, Trento, Italy; 3 Leibniz Institute for Age Research – Fritz Lipmann Institute, Jena, Germany

**Keywords:** Noggin, BMP, Cortical identity, Embryonic stem cells

## Abstract

We investigated the effects of bone morphogenetic proteins (BMPs) in determining the positional identity of neurons generated in vitro from mouse embryonic stem cells (ESCs), an aspect that has been neglected thus far. Classical embryological studies in lower vertebrates indicate that BMPs inhibit the default fate of pluripotent embryonic cells, which is both neural and anterior. Moreover, mammalian ESCs generate neurons more efficiently when cultured in a minimal medium containing BMP inhibitors. In this paper, we show that mouse ESCs produce, secrete, and respond to BMPs during in vitro neural differentiation. After neuralization in a minimal medium, differentiated ESCs show a gene expression profile consistent with a midbrain identity, as evaluated by the analysis of a number of markers of anterior–posterior and dorsoventral identity. We found that BMPs endogenously produced during neural differentiation mainly act by inhibiting the expression of a telencephalic gene profile, which was revealed by the treatment with Noggin or with other BMP inhibitors. To better characterize the effect of BMPs on positional fate, we compared the global gene expression profiles of differentiated ESCs with those of embryonic forebrain, midbrain, and hindbrain. Both Noggin and retinoic acid (RA) support neuronal differentiation of ESCs, but they show different effects on their positional identity: whereas RA supports the typical gene expression profile of hindbrain neurons, Noggin induces a profile characteristic of dorsal telencephalic neurons. Our findings show that endogenously produced BMPs affect the positional identity of the neurons that ESCs spontaneously generate when differentiating in vitro in a minimal medium. The data also support the existence of an intrinsic program of neuronal differentiation with dorsal telencephalic identity. Our method of ESC neuralization allows for fast differentiation of neural cells via the same signals found during in vivo embryonic development and for the acquisition of cortical identity by the inhibition of BMP alone.

**Electronic supplementary material:**

The online version of this article (doi:10.1007/s00018-012-1182-3) contains supplementary material, which is available to authorized users.

## Introduction

Neural inducing signals are proposed to impart both neural and anterior identity to the ectoderm, while the generation of the full range of CNS structures would be the result of later events that posteriorize anterior neural tissue. According to this view, bone morphogenetic proteins (BMPs) play a key role by antagonizing a neural anterior default differentiation program. Antagonists of BMP signaling such as Noggin would ensure low levels of BMPs in the presumptive neuroectoderm thus allowing forebrain development in the absence of posteriorizing signals [67].

The dissection of diffusible signals that orchestrate neural induction has recently been made easier by the study of embryonic stem cells (ESCs) in vitro differentiation. In recent years, several reports have described methods for the generation of neural cells from mouse ESCs [5, 15, 19, 65, 66]. Using defined growth media, it has been possible to investigate the diffusible factors that affect anterior–posterior (A/P) as well as dorsoventral (D/V) identity of in vitro-generated neurons. Among these, retinoic acid (RA), BMPs, Wnts, fibroblast growth factors (FGFs) and sonic hedgehog (SHH) have been described [10, 15, 18, 28, 65].

Conversely, the use of factor-free chemically defined media has allowed for the investigation of the differentiation fate of ESCs in the absence of exogenous signals, showing that it is predominantly neural [19, 20, 63]. Effects of factors endogenously produced by ESCs have also been suggested. BMPs sustain self-renewal and inhibit neural differentiation of ESCs [70]. The BMP inhibitor Noggin triggers in vitro neuronal differentiation of mammalian ESCs cultured in growth factor-free chemically defined medium [9, 22]. It was recently shown that the cell-intrinsic expression of the zinc-finger nuclear protein Zfp521, which is inhibited by BMPs, plays a pivotal role in promoting a default neural state of ESCs. Furthermore, a role of Zfp521 in supporting an anterior identity of neurons generated by ESCs was hypothesized [33]. These data suggest that ESCs produce and are sensitive to BMPs with an autocrine/paracrine mechanism. However, to our knowledge, there is no direct measurement of BMP production by differentiating ESCs.

Early studies in lower vertebrates suggested that BMP plays a key role in anterior/posterior patterning. BMP antagonism on pluripotent cells of *Xenopus* animal caps induces cement glands, which are the most anterior ectodermal structures in *Xenopus*, and anterior brain markers such as the fore-midbrain marker Otx2, but not hindbrain or spinal cord markers [26, 38, 56]. More recent studies highlight that Noggin has a dose-dependent patterning effect on *Xenopus* animal caps. At lower doses, Noggin supports neuralization without the expression of diencephalic markers, which are instead activated at higher doses [39]. Moreover, in *Xenopus* embryos, the specification of the forebrain requires isolation of its cells from BMP, Activin/Nodal, and Wnt signaling by high concentrations of Noggin produced in cells at the anterior margin of the neural plate [4]. These observations suggest that, in vivo, the concentration of endogenous BMPs might be relevant in the control of the positional identity of neurons. It has also been proposed that BMPs play a role in the regional morphogenesis of mouse dorsal telencephalon, by the control of specific gene expression, cell proliferation, and local cell death [17]. Forebrain truncations were found in double-mutant mice for both BMP antagonists Noggin and Chordin [3]. BMP signaling specifies telencephalic progenitor cells toward the most dorsal fate, the choroid plexus [27], but earlier effects on the anterior–posterior patterning are not well characterized.

The aim of the present work is to directly show the endogenous production of BMP by differentiating ESCs and to characterize the effects of BMP on the differentiation and positional identity of ESC-generated neurons. We, therefore, established an in vitro differentiation protocol that minimizes exogenous signals and analyzed ESCs differentiation by performing a genome-wide expression analysis. We report that mouse ESCs produce and release BMPs, which act on their differentiation in such a minimal medium. Blocking the BMP pathway by Noggin or by other inhibitors selectively affects the A/P positional identity of the generated neurons. At the highest doses of Noggin that we tested, the fate of neurons produced by ESCs is predominantly dorsal telencephalic. These neurons have a gene expression profile that clusters with that of early cerebral cortical cells and express telencephalic differentiation markers.

## Materials and methods

### Cells cultures

Murine embryonic stem cell (ESC) lines E14Tg2A (passages 25–38) and 46 C (transgenic Sox1-GFP ESC kindly provided by A. Smith, University of Cambridge, UK, passages 33–39) were cultured on gelatin-coated tissue culture dishes at a density of 40,000 cells/cm^2^. ESC medium, which was changed daily, contained GMEM (Sigma), 10 % Fetal Calf Serum, 2 mM Glutamine, 1 mM sodium Pyruvate, 1 mM non-essential amino acids, 0.05 mM *β*-mercaptoethanol, 100 U/ml Penicillin/Streptomycin and 1,000 U/ml recombinant mouse LIF (Invitrogen). Cells were passaged using Trypsin dissociation and re-plated at a dilution of 1:3–1:4, to avoid cell confluence and to maintain pluripotency. RAW 264.7 (mouse leukemic monocyte macrophage cell line, kindly supplied by Diana Boraschi, Institute of Medical Biotechnology, CNR of Pisa) were cultured in Dulbecco’s modified Eagle’s Medium with 4 mM l-glutamine and 4.5 g/L glucose, supplemented with 10 % fetal bovine serum. Cells were split every 2 days at a confluence of approximately 10 % (1 × 10^6^ and 3 × 10^6^ cells in 100- and 150-mm plates, respectively) and grown to a confluence of approximately 80 %. Mouse mesenchymal stromal cells (MSCs) primary cultures (kindly supplied by Cristina Magli, CNR of Pisa) were established from B6D2F1 (BDF1) mice (Charles River) as described [44].

### Neural induction

Chemically defined minimal medium (CDMM) consisted of DMEM/F12 (Invitrogen), 2 mM Glutamine, 1 mM sodium Pyruvate, 0.1 mM non-essential amino acids, 0.05 mM β-mercaptoethanol, 100 U/ml Penicillin/Streptomycin supplemented with N2/B27 (no vitamin A; Invitrogen). Step I: dissociated ESCs were washed with DMEM/F12, aggregated in agar-coated culture dishes (65,000 cells per cm^2^) and cultured as floating aggregates in CDMM for 2 days. The second day 70 % of CDMM was renewed. Step II: ESCs aggregates were dissociated and cultured in adhesion (65,000 cells per cm^2^) on Poly-ornithine (Sigma; 20 μg/ml in sterile water, 4 h coating at 37 °C) and natural mouse Laminin (Invitrogen; 5 μg/ml in PBS, 4 h coating at 37 °C) for 4 days, changing CDMM daily. Step III: After a second dissociation, ESCs were cultured 4 additional days in CDMM devoid of B27 supplement to drive terminal differentiation, using the same type of seeding density and coated surface. Serum employed for trypsin inactivation was carefully removed by several washes in DMEM/F12. The following factors were tested by addition during step II: Recombinant Mouse Noggin (R&D; ranging from 5 to 400 nM), BMP4 (R&D; 50 ng/ml), Recombinant Mouse BMPRIA/Fc Chimera (R&D; 3.75 and 37.5 nM), Dorsomorphin (Sigma-Aldrich; 5 μM), Retinoic Acid (Sigma-Aldrich; 0.1–10 μM), Cyclopamine (Sigma-Aldrich; 10 μM), SAG (Santa Cruz Biotechnology; 100 nM), SB431542 (Sigma-Aldrich; 10 μM). Cell viability and proliferation, which were monitored by trypan blue exclusion test and cell counting, respectively, were not significantly affected by treatments.

### Semiquantitative real-time PCR

For each sample, 500 ng of total RNA were reverse-transcribed. Amplified cDNA was quantified using GoTaq qPCR Master Mix (Promega) on Rotor-Gene 6000 (Corbett) with the primers listed in Supplemental Table 1. Amplification take-off values were evaluated using the built-in Rotor-Gene 6000 “relative quantitation analysis” function, and relative expression was calculated with the 2^−*ΔΔ*Ct^ method, normalizing to the housekeeping gene *β*-Actin. Standard errors were obtained from the error propagation formula as described in [46], and statistical significance was probed with randomization test, taking advantage of REST Software [51].

### Immunocytodetection

Cells prepared for immunocytodetection experiments were cultured on Poly-ornithine/Laminin coated round glass coverslips. Cells were fixed using 2 % paraformaldehyde for 15 min, washed twice with PBS, permeabilized using 0.1 % Triton X100 in PBS and blocked using 0.5 % BSA in PBS. Primary antibodies used for microscopy included Oct3/4 (1:200; Santa Cruz DBA), Nanog (1:300; Novus Biologicals), acetylated N-Tubulin (1:500; Sigma), Neuronal Class III β-Tubulin (1:500; Covance), Doublecortin (1:500; Abcam), Musashi-1 (1:200; Cell Signaling), Nestin (1:200; Millipore), Synaptophysin (1:100; Santa Cruz DBA), α-Internexin (1:100; Santa Cruz DBA), phospho-Smad1/5/8 (1:100; Millipore), FoxG1 (1:200; Abcam), Tbr1 (1:400; Millipore), Satb2 (1:200; Abcam), VGlut2 (1:300; Abcam), GAD65 (1:500; Chemicon), Pax6 (1:400; Covance), Nkx2.1 (1:400; Abcam) and GFAP (1:100; Dako). Primary antibodies were incubated 2 h at room temperature; cells were then washed three times with PBS (10′ each). Alexa Fluor 488 and Alexa Fluor 568 anti-mouse or anti-rabbit IgG conjugates (Molecular Probes, 1:500) were incubated 1 h at RT in PBS containing 0.1 % Triton X100 and 0.5 % BSA for primary antibody detection, followed by three PBS washes (10′ each). Nuclear staining was obtained with DAPI. The protocol varied for Tbr1, Satb2 and FoxG1, the antibodies of which were incubated overnight at 4 °C using 0.3 % Triton X100.

### FACS analysis

Adherent cells were detached by trypsinization, washed and resuspended in PBS at RT, then analyzed with a FACSCalibur cytometer (BD). At least 10,000 events per sample were collected. Data were processed with the free software WinMDI 2.9 (The Scripps Research Institute).

### BMP2 ELISA

Cells were seeded into 24-well plates and cultured as described. When cells reached 70–80 % confluence, each well was washed with PBS and fresh medium (DMEM/F12 containing 2 mM Glutamine and 1 mM sodium Pyruvate) was replaced. After 24 h, supernatant was collected, centrifuged (10,000*g*, 5 min) to remove particulates and assayed for BMP2 content with a commercially available ELISA kit (Quantikine, BMP-2 Immunoassay; R&D Systems, Minneapolis, MN, USA), according to the manufacturer’s instructions. This assay could measure BMP-2 concentrations as low as 50 pg/ml in a linear range (Pearson correlation coefficient of linear regression *R* ^2^ = 0.999; see Supplemental Figure SF1). A 1.2 % cross-reactivity was observed with 50 ng/mL recombinant human BMP-4. BMP-2 concentrations of triplicate samples were determined from the optical densities at 450 nm in relation to standard curves of the recombinant antigen provided in the kit.

### Microarray hybridization and data analysis

Cortex, midbrain and hindbrain were dissected from *n* = 3 mouse embryos (C57BL/6 strain) at embryonic day (E)16. Total RNA was extracted with NucleoSpin RNA II columns (Macherey–Nagel). RNA from three different sets of experiment was pooled. RNA quality was assessed with Agilent Bioanalyzer RNA 6000 Nano kit; 500 ng of RNA were labeled with One Color Quick amp labeling kit (Agilent), purified and hybridized overnight onto an Agilent Mouse Gene Expression Microarray chip (4 × 44 Kv2) before detection, according to the manufacturer’s instructions. Three slides were hybridized with Noggin-treated ESCs RNA and two slides were hybridized with RNA from all the other conditions. Agilent DNA Microarray scanner was used for slide acquisition and spot analysis was performed with Feature Extraction software (Agilent).

For GSEA analysis, genes differentially expressed between Noggin treatment and CDMM (Supplemental Table 2), or between RA treatment and CDMM (Supplemental Table 3; fold-change ≥2), were analyzed by the GeneSpring GX11.0 software using BROAD Gene Ontology collection (C5; http://www.broadinstitute.org/gsea). A complete GSEA list with enrichment scores of gene sets with *q* value <0.3 is shown in Supplemental Table 4.

To select a gene set representing the anterior-posterior regionalization of the developing brain, we compared gene expression profiles of E16 cortex and hindbrain using Genespring GX11.0 software (Agilent). A set of 592 genes with an absolute fold-change greater or equal than 10 (*p* < 0.05) was selected (see Supplemental Table 5). Significance of the data was proven by one-way ANOVA and Tukey post hoc test with Bonferroni correction for multiple comparisons. The content of this set of genes was explored by hierarchical clustering and principal component analysis, taking advantage of Cluster software [16]. Single linkage algorithm was employed for hierarchical clustering. Trees were generated using absolute correlation for genes and Euclidean distance for arrays, and visualized with java TreeView [55].

## Results

### A chemically defined minimal medium (CDMM) induces neurogenesis of ESCs

**Fig. 1:**
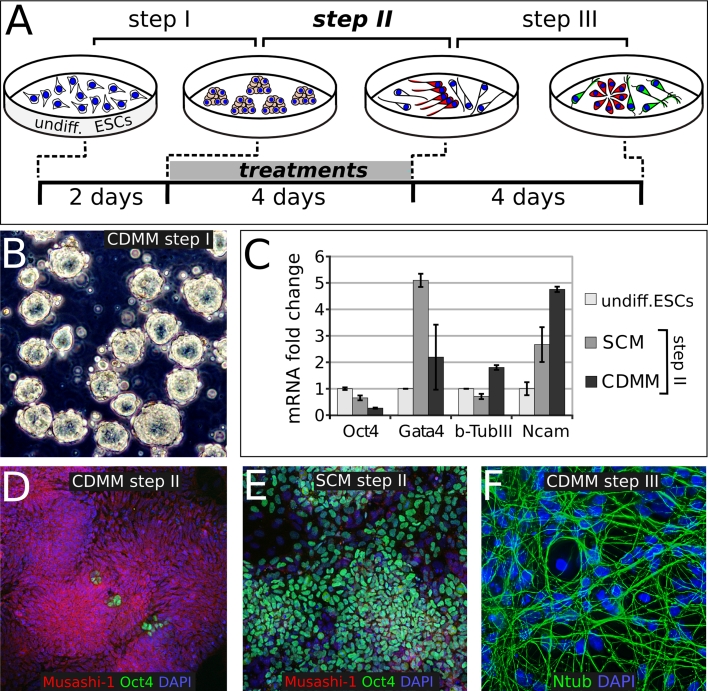
Three-step protocol of ESCs neuronal differentiation: **a** ESCs differentiation protocol outline; *undiff* undifferentiated. **b** ESCs aggregates at step I. **c** RT-PCR mRNA analysis of expanding ESCs (undifferentiated), or of ESCs at the end of step II, initially (step I) aggregated in two diverse conditions (SCM and CDMM), normalized on ESCs. **d**, **e** Oct4 (*green*) and Musashi-1 (*red*) immunocytodetection of ESCs at the end of step II, after CDMM (**d**) or SCM (**e**) aggregation in step I. **f** immunocytodetection for anti-acetylated-Tubulin antibody (Ntub, *green*) of ESCs aggregated and differentiated in CDMM, at the end of step III. *Error bars* standard error; *p* < 0.001 (Randomization test, REST software) for all SCM and CDMM values compared to ESCs values, except for Gata4 in CDMM, which was not significant

In order to investigate the default positional identity of neurons generated from ESCs, we established a culture method that promotes neurogenesis minimizing the influence of exogenous signals. This method consists of a three-step procedure of culture in a chemically defined minimal medium (CDMM; see “Materials and methods”; Fig. 1a) devoid of serum or morphogens but allowing cell survival by insulin.

Upon LIF and serum withdrawal, dissociated ESCs were initially grown as aggregates (Fig. 1b) in CDMM for 2 days. This step (step I), which minimizes cell death, follows a procedure adapted from previously described methods [5, 40, 65]. As many protocols use serum-containing medium (SCM) during ESCs aggregation, we also assayed this condition in the preliminary set-up of our protocol. As additional control, we used undifferentiated ESCs cultured in LIF + serum (ESC medium).

ESC aggregates were subsequently dissociated and cultured in adhesion for 4 days on Poly-ornhitine/Laminin-coated wells in CDMM (step II). All additional treatments (e.g., Noggin), when applied, were performed during this step (Fig. 1a), unless specified. During step II, ESCs turned off the expression of the stem cell marker Oct4 [45] and activated the expression of the pan-neuronal markers β-Tubulin-III and Ncam, as seen by RT-PCR (Fig. 1c). This activation was higher in ESCs aggregated in CDMM than in ESCs aggregated in SCM, as the latter still expressed high levels of Oct4 and activated the mesodermal marker GATA4 (Fig. 1c). Immunostaining showed much higher expression of the neural progenitor cell marker Musashi-1 [47] and robust downregulation of Oct4 in ESCs cultured in CDMM (Fig. 1d) compared to ESCs cultured in SCM (Fig. 1e). Whereas ESCs aggregated in CDMM started expressing β-Tubulin-III at step II, ESCs aggregated in SCM failed to show β-Tubulin-III labeling (Supplemental Figure SF2A, B). Similar results were obtained when analyzing ESCs aggregates cultured for 5 days (Supplemental Figure SF2C, D). Our observations indicate that aggregation (step I) in the absence of serum facilitates loss of stem cell pluripotency and induces rapid neural differentiation (as evaluated at the end of step II).

After a second dissociation, cells were cultured for 4 days in CDMM. This additional step (step III) allowed cells to undergo terminal differentiation. Notably, the presence of serum during step I profoundly affected the fate of cells produced at the end of step III, as the ratio of neural progenitor cells immunostained by Nestin antibody was lower in cells aggregated in SCM (8 ± 4.8 %;) compared to cells aggregated in CDMM (44.3 ± 8.7 %; Supplemental Figure SF2E–G). Consistently, mRNA expression of Nestin and of pan-neuronal markers Ncam and β-Tubulin-III was significantly lower at the end of step III in cells that were aggregated in SCM than in cells that were aggregated in CDMM (Supplemental Figure SF2H). Moreover, cells cultured in CDMM formed rosette-like structures at the end of step III, which are typical of neural progenitors in vitro ([73]; Supplemental Figure SF2F), and generated high proportions of neuronal cells immunostained by anti-acetylated-Tubulin (Ntub, Fig. 1f) and β-Tubulin-III antibody (Supplemental Figure SF2I).

We further characterized the nature of the differentiated ESCs by immunocytodetection. Neuronal morphology was heterogeneous, as we found multipolar cells, pyramidal-like cells, bipolar and unipolar cells (Supplemental Figure SF2J–M). ESC-generated neurons showed processes with varicosities positive to the neuronal intermediate filament α-Internexin that are typical of neurons forming synapses (Supplemental Figure SF2N). Moreover, ESC-derived neurons showed a punctate staining of the synaptic marker Synaptophysin (Supplemental Figure SF2O). We failed to detect GFAP-positive cells by immunostaining and GFAP mRNA levels were very low compared to the levels of P0 embryonic cortex, as evaluated by RT-PCR (not shown). This is consistent with an early differentiation state of the cells, as gliogenesis is the latest step in ESCs neural differentiation protocols [19].

We concluded that a short exposure to serum in the first days of differentiation cultures (step I) inhibits the acquisition of a neuronal fate and that ESCs cultured in the absence of any added morphogen efficiently differentiate into neuronal cells, which is consistent with previous observations [19, 59].

### Effects of Noggin as a neural inducer in ESCs culture

**Fig. 2:**
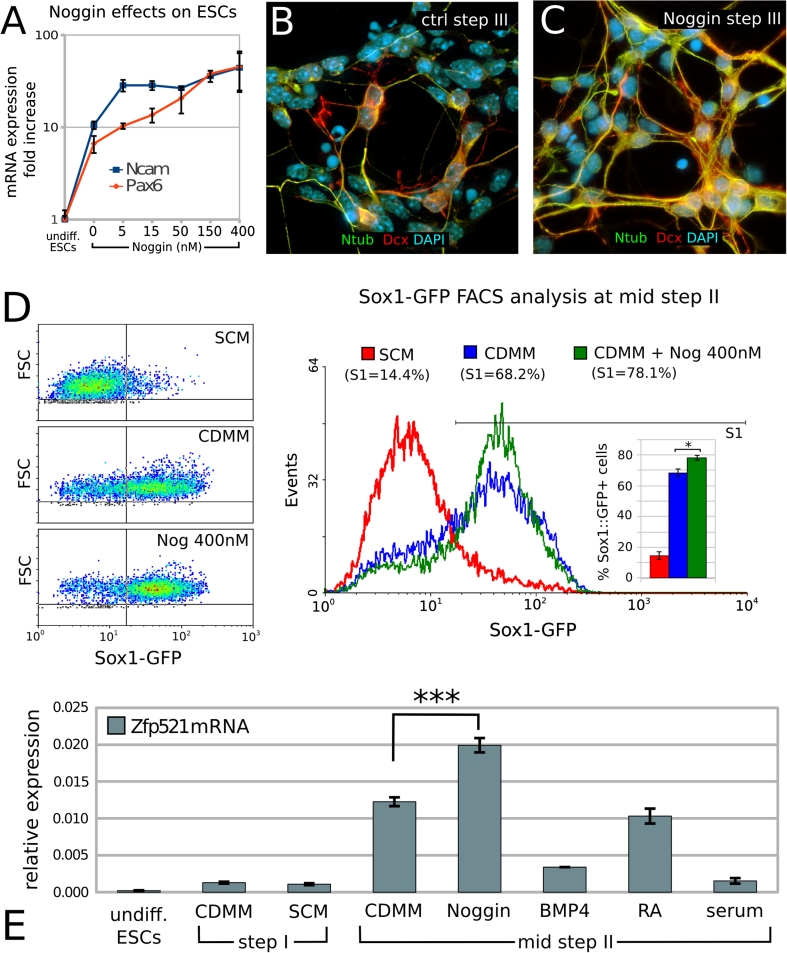
Effects of Noggin as a neural inducer on ESCs differentiation:**a** RT-PCR mRNA quantification of pan-neuronal markers Ncam and Pax6 in expanding ESCs (undifferentiated) and ESCs at the end of step III after differentiation in CDMM (0) or in CDMM plus 5–400 nM Noggin (expression normalized on undifferentiated ESCs). **b**, **c** Doublecortin (Dcx, *red*) and acetylated-Tubulin (Ntub, *green*) immunocytodetection of ESCs at the end of step III after differentiation in CDMM (**b**, ctrl) or in CDMM containing 150 nM Noggin (**c**). **d** Flow-cytometry analysis of Sox1-GFP ESCs at day 2 of step II after culture in different conditions. SCM: ESCs were aggregated (step I) in serum-containing medium and differentiated (2 days, step II) without serum. CDMM: both ESCs aggregation (step I) and differentiation (2 days, step II) were carried out without serum. CDMM + Nog 400 nM: as CDMM condition, plus Noggin treatment during the first two days of step II. **e** RT-PCR mRNA quantification (ratio over β-Actin) of Zfp521 at different time points and after culture in different conditions, as indicated; treatments with Noggin (400 nM), BMP4 (50 ng/ml), RA (10 nM) and serum (10 %) were performed at step II. In (**a**, **d**, **e**), *error bars* show standard error; *p < 0.05, ***p < 0.001 (two-tailed Student’s *t* test)

The ability of Noggin to support neuronal differentiation of ESCs has been reported in different in vitro differentiation protocols [12, 22, 43]. Consistently, we found that adding increasing doses of Noggin (5–400 nM) to CDMM during step II supported the expression of the pan-neuronal markers Ncam and Pax6, as compared to cells grown in CDMM without Noggin (Fig. 2a). Notably, Noggin did not significantly affect pan-neuronal markers expression when added at step I or III (not shown).

At the end of step III, ESCs treated with Noggin during step II slightly increased the expression of Doublecortin (Dcx, a general marker of migrating neuroblasts; [36]) and of acetylated-Tubulin (Ntub, pan-neuronal marker) compared to ESCs cultured in CDMM (ctrl; Fig. 2b, c).

We directly compared the neural inducing activity of Noggin to SCM and CDMM conditions in terms of percentage of neural progenitors generated by ESCs. We took advantage of a Sox1-GFP knock-in ES cell line (46 C, [70]), analyzing GFP expression by flow cytometry. This analysis showed that CDMM culture conditions induced a massive increase of Sox1-GFP-positive neural progenitors at mid-step II (day 2; 68.2 %) compared to SCM condition (14.4 %), whereas Noggin (400 nM) induced a modest, although significant, increase compared to CDMM (78.1 %; Fig. 2d). A similar trend was also observed at early-step II (day1; SCM, 6.4 %; CDMM, 27.9 %; Noggin, 31.1 %; not shown). Ratios of Sox1-GFP positive neural progenitors obtained in the different culture conditions are consistent with a differential expression of the key transcription factor of neural commitment Zfp521, which is highest in cultures with Noggin (400 nM; Fig. 2e). We concluded that the majority of ESCs cultured in CDMM or in CDMM plus Noggin become neural progenitors at step II and established CDMM culture condition as control for subsequent investigations on BMP inhibition.

### CDMM-differentiating ESCs produce and respond to BMPs

**Fig. 3:**
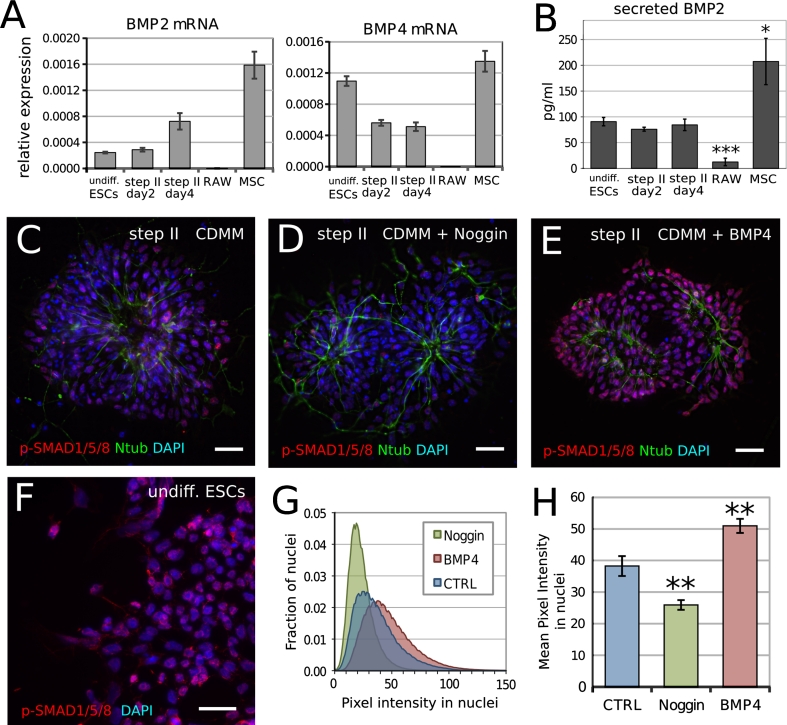
Endogenous BMP production and BMP activity during ESCs differentiation in CDMM: **a** RT-PCR mRNA quantification (ratio over β-Actin) of BMP2 and BMP4 in expanding ESCs (undifferentiated), ESCs at the second and fourth day of step II, expanding RAW 264.7 cell line (RAW) and mouse mesenchymal stromal cells (MSCs, passage 3). **b** Secreted BMP2 quantification by ELISA in cells as in (**a**). **c–e** Ntub (*green*) and phospho-SMAD1/5/8 immunodetection (nuclear red staining over DAPI nuclear counterstaining) in ESCs at step II (day 2) in CDMM (**c**), 5 h after the addition of 400 nM Noggin to CDMM (**d**) and 5 h after the addition of 50 ng/ml BMP4 to CDMM (**e**). **f** Phospho-SMAD1/5/8 immunodetection (*red* staining over DAPI) in undifferentiated ESCs. *Scale bars* 30 μm. **g, h** Pixel intensity distribution (fraction of nuclei with given pixel intensity (**g**) and mean pixel intensity (**h**) of immunodetection in nuclei as in (**c–e**), respectively. *Error bars* standard error; *p < 0.05, **p < 0.01, ***p < 0.001 (Student’s *t* test)

As Noggin affects ESCs neuralization, but BMPs were not added to culture medium, we assayed for the presence of BMPs that were endogenously produced by ESCs during differentiation. We thus compared the mRNA expression levels of BMP2/4 in proliferating ESCs, in CDMM-differentiating ESCs (during step II), in cells that express high BMP levels (primary mouse mesenchymal stromal cells, MSCs) or in cells that express low BMP2/4 levels (macrophage cell line RAW 264.7; [52]). We found that both undifferentiated and differentiating ESCs express high BMP2/4 levels (Fig. 3a). Notably, ESCs transcribe BMP2/4 also at step II, when Noggin addition to CDMM exerts its effect on neuronal differentiation. Moreover, ELISA showed that CDMM-differentiating ESCs secrete approximately 50 % of the BMP2 protein secreted by MSCs, but almost ten times more than the amount secreted by RAW cells (Fig. 3b).

We found that CDMM-differentiating ESCs during step II express functional BMP receptors. In fact, ESCs at step II expressed higher levels of BMPR1a-b mRNA than MSCs, which depend on the binding of BMP2/4 to BMPR1a-b receptors for osteoblastogenesis [1] (Supplemental Figure SF3A). Interestingly, Noggin decreased the expression of ID1, a downstream effector of the BMP-responsive pathway [30] in a dose-dependent manner (Supplemental Figure SF3B). This is in line with the ability of noggin to block the BMP-responsive pathway in ESCs.

We further investigated the activation of intracellular transduction pathway in response to BMP signaling by analyzing SMAD1/5/8 phosphorylation (phospho-SMAD; Fig. 3c–h). Undifferentiated ESCs were used as a positive control (Fig. 3f). Most nuclei of both undifferentiated ESCs and ESCs at step II in different conditions showed phospho-SMAD immunostaining, with different degrees of intensity. Figure 3g shows the distribution of the immunostaining intensity and Fig. 3h reports the mean immunostaining intensity. We found that control CDMM-differentiating ESCs show intermediate levels of the phosphorylated form of SMAD1/5/8 during step II, as compared to ESCs in other culture conditions (Fig. 3c). This confirms the presence of an endogenous BMP production and activity. Acute 5 h treatment with Noggin (400 nM) or BMP4 (50 ng/ml) during step II significantly decreased or increased SMAD phosphorylation, respectively (Fig. 3d–g). The pattern of phospho-SMAD immunodetection showed that virtually all cells responded to BMP4 addition (Fig. 3e).

Consistently, BMP4 added exogenously to CDMM throughout step II (50 ng/ml), dramatically repressed the expression of the pan-neuronal markers Nestin, NFL, β-Tubulin-III and Pax6 (Supplemental Figure SF3C), thus confirming the ability of ESCs to specifically respond to BMP signaling during step II.

Our data thus show that ESCs produce and are sensitive to BMPs during neuronal differentiation in vitro.

### In the absence of exogenous signals, ESCs generate neurons expressing midbrain dorsal markers

**Fig. 4:**
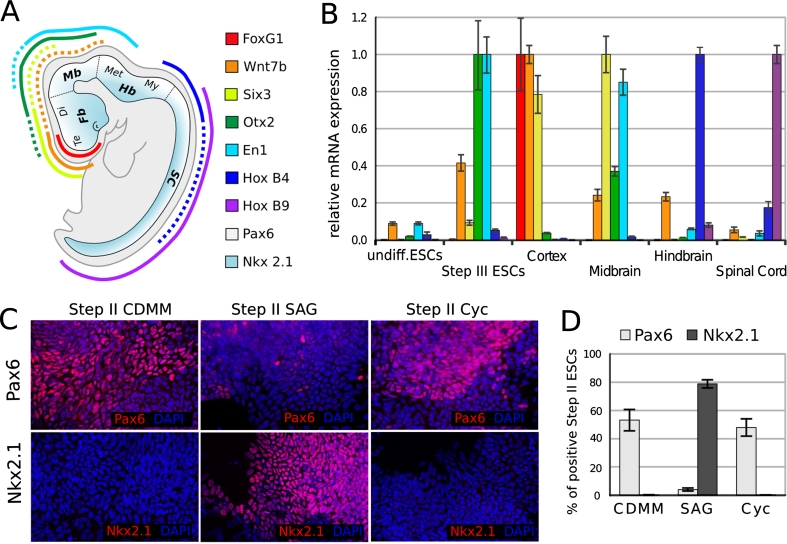
Regional identity of ESCs differentiated in CDMM: **a** A/P (*color code*) and D/V (*white-cyan code*) patterning of mouse embryo as identified by the expression of key patterning genes, elaborated from EMAP (http://www.emouseatlas.org/emap/home.html) and articles cited in text. *Fb* forebrain, *Mb* midbrain, *Hb* hindbrain, *SC* spinal cord, *Te* telencephalon, *Di* diencephalon, *Met* metencephalon, *My* myelencephalon. **b** mRNA relative expression of A/P genes (as evaluated by RT-PCR, normalized on maximum expression) in brain tissues of E16 embryos, undifferentiated ESCs and ESCs at the end of step III. **c** Immunocytodetection of Pax6 and Nkx2.1 (nuclear *red* staining over DAPI nuclear counterstaining) at the end of step II in ESCs differentiated as indicated. Numbers in (**d**) show fractions of Pax6-positive cells (*light gray bars*) and Nkx2.1-positive cells (*dark gray bars*), in ESCs differentiated as in (**c**). *Cyc* cyclopamine. *Error bars* standard error

In order to investigate the effect of endogenous BMPs on ESCs positional identity, we characterized our control culture (ESCs differentiated in CDMM), by analyzing the expression of the FoxG1 [69], Wnt7b [49], Six3 [48], Otx2 [2], and En1 [68] genes at the end of step III. These genes display an ordered (A/P) expression that covers the most anterior aspect of forebrain (FoxG1, Emx2), entire forebrain (Six3), forebrain/midbrain (Otx2), and midbrain (En1). We also analyzed the expression of HoxB4 [53] and HoxB9 [11], which mark hindbrain and spinal cord, respectively (Fig. 4a). We compared the mRNA levels of these genes in CDMM-differentiated ESCs to the mRNA levels found in cortex (rostral–dorsal forebrain), mesencephalon (midbrain), rombencephalon (hindbrain), spinal cord of embryonic day 16 (E16) mouse, and undifferentiated ESCs. Compared to mouse brain, CDMM-differentiated ESCs expressed very high levels of Otx2 and En1, low levels of Wnt7b and Six3, and very low levels of both telencephalic (FoxG1), and posterior markers (HoxB4 and HoxB9) (Fig. 4b).

As ESCs cultured in CDMM failed to express high levels of hindbrain/spinal cord specific genes, we wanted to assay their ability to turn on these genes upon induction with the posteriorizing factor RA. As expected, ESCs treated with RA during step II, and analyzed at the end of step III, turned on the posterior markers HoxB4 and HoxB9 and downregulated the anterior markers FoxG1, Six3 and Otx2 in a dose-dependent fashion (Supplemental Figure SF4A).

We then analyzed the dorso-ventral (D/V) identity of ESCs generated cells at the end of step II by comparing the relative ratios of the cells expressing the dorsal marker Pax6 [60] or the ventral marker Nkx2.1 [50]. A large fraction (53.1 ± 7.6 %) of control CDMM ESCs expressed Pax6 protein and virtually no cells expressed Nkx2.1 protein (Fig. 4c, d). As the Pax6/Nkx2.1 D/V gradient is generated in response to a gradient of sonic hedgehog (Shh) activity [7], we assayed the effects of a SHH agonist (SAG, [13]) or of an antagonist (Cyclopamine; [62]) on ESCs. Drugs were added to CDMM throughout step II. SAG treatment dramatically repressed Pax6 (3.9 ± 1.1 %) and activated Nkx2.1 protein expression in a very large fraction of cells (79 ± 2.9 %), whereas Cyclopamine affected neither Pax6 nor Nkx2.1 (Fig. 4c, d). RT-PCR analysis confirmed these results (Supplemental Figure SF4B). Notably, the lack of any effect of Cyclopamine is consistent with the observation that Step II ESCs produced very low level of endogenous SHH (Supplemental Figure SF4C).

These data suggest that in our protocol of differentiation ESCs change the expression of A/P and D/V markers accordingly to treatments with morphogens, but mostly adopt a midbrain dorsal identity when cultured in CDMM (see “Discussion”).

### BMP inhibition during differentiation supports the expression of telencephalic markers

**Fig. 5:**
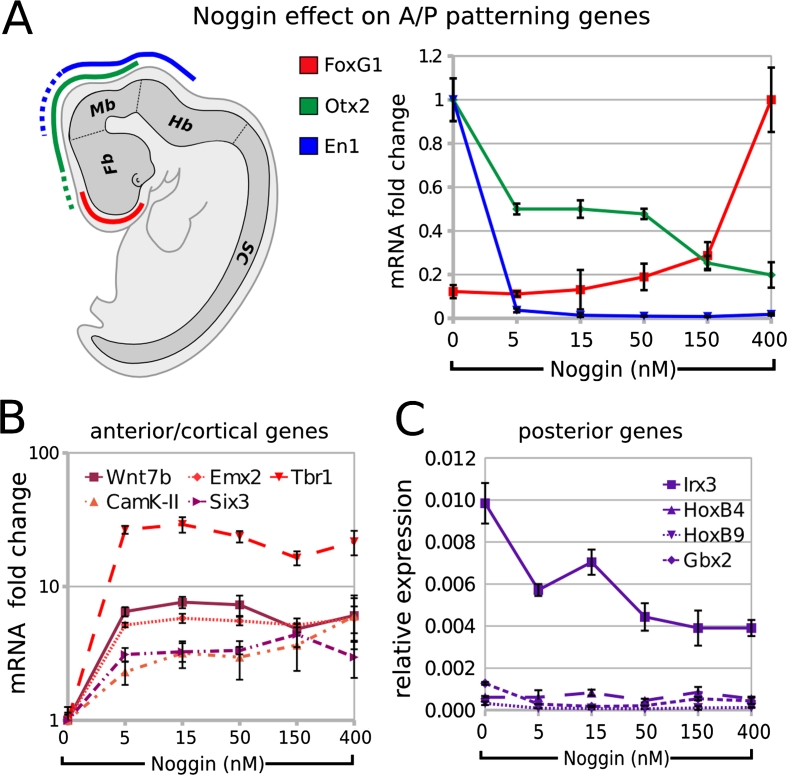
Effects of BMP inhibition on the expression of A/P patterning genes: **a** A/P (*color code*) patterning of mouse embryonic brain by FoxG1, Otx2 and En1. *Fb* forebrain, *Mb* midbrain, *Hb* hindbrain, *SC* spinal cord. Graph shows RT-PCR mRNA quantification of FoxG1, Otx2 and En1 in ESCs at the end of step III after differentiation in CDMM (0) or in CDMM plus 5–400 nM Noggin (normalized on maximum expression). **b**,** c** RT-PCR mRNA quantification of forebrain/cortical markers (**b**) or hindbrain/spinal cord markers (**c**), in cells as in A (ratio over β-Actin). *Error bars* standard error

We subsequently investigated if endogenously produced BMPs can affect the regional identity of ESC-generated neurons. Compared to control, the treatment with increasing doses of Noggin (5–400 nM) during step II induced the telencephalic marker FoxG1 and repressed the more posterior markers Otx2 and En1 (Fig. 5a) in a dose-dependent manner, as evaluated at the end of step III. Moreover, Noggin induced the expression of Wnt7b (a forebrain marker; [49]), Six3 (prosencephalic marker), Emx2 (early cortical marker; [58]), Tbr1 and α-CamK-II (late cortical markers; [8]; [35]; Fig. 5b), and repressed the expression of the posterior marker Irx3, which is present in midbrain and more posterior regions ([6]; Fig. 5c). Noggin was ineffective on the hindbrain/spinal cord markers Gbx2, HoxB4 and HoxB9, leaving their low expression levels almost unchanged (Fig. 5c). As similar results were obtained when analyzing cells at earlier or later times of differentiation (end of step II or step III plus 4 days, respectively; Supplemental Fig. 5), we excluded the possibility that the effect of Noggin on positional identity may be the result of an enhancement/acceleration in neural fate induction.

To confirm the specificity of action of Noggin, we used the chimeric protein BMPR1A-Fc, a BMP inhibitor that binds to a BMP epitope outside the region recognized by Noggin ([23, 34]; see Supplemental Figure SF6A) and Dorsomorphin, a selective inhibitor of the BMP type I receptors ALK2, ALK3 and ALK6 that blocks BMP-mediated SMAD1/5/8 phosphorylation ([72]; Supplemental Figure SF6B). BMPR1A-Fc induced an increase of Sox1-GFP positive neural progenitors at mid-step II (day 2; 77.6 %; Supplemental Figure SF6D) that is comparable to the increase induced by Noggin (78.1 %; Fig. 2d). Dorsomorphin or BMPR1A-Fc treatment during step II also mimicked Noggin action by inhibiting ID1 expression (Supplemental Figures SF3B and SF6C), by supporting FoxG1 expression and by repressing Otx2 and En1 (Supplemental Figure SF6E, F). The specificity of BMPs in affecting ESCs differentiation fate is also suggested by the effects exerted by treatment at step II with SB431542. While Dorsomorphin selectively inhibits the BMP2/4 pathway, SB431542 suppresses the Activin/Tgf-β receptors ALK4, ALK5 and ALK7 and prevents BMP-independent, Activin/Tgf-β mediated, SMAD2/3 phosphorylation (Supplemental Figure SF6B; [9]). Compared to Noggin and Dorsomorphin, SB451243 acted by repressing, rather than by inducing, FoxG1, slightly inhibited En1 and left Otx2 expression almost unchanged (Supplemental Figure SF6E).

Our results indicate that the inhibition of endogenously produced BMPs alters the A/P positional identity of the ESC-generated neurons.

### BMP inhibition induces a mixed population of neural progenitor cells and differentiated neurons expressing cortical markers

**Fig. 6:**
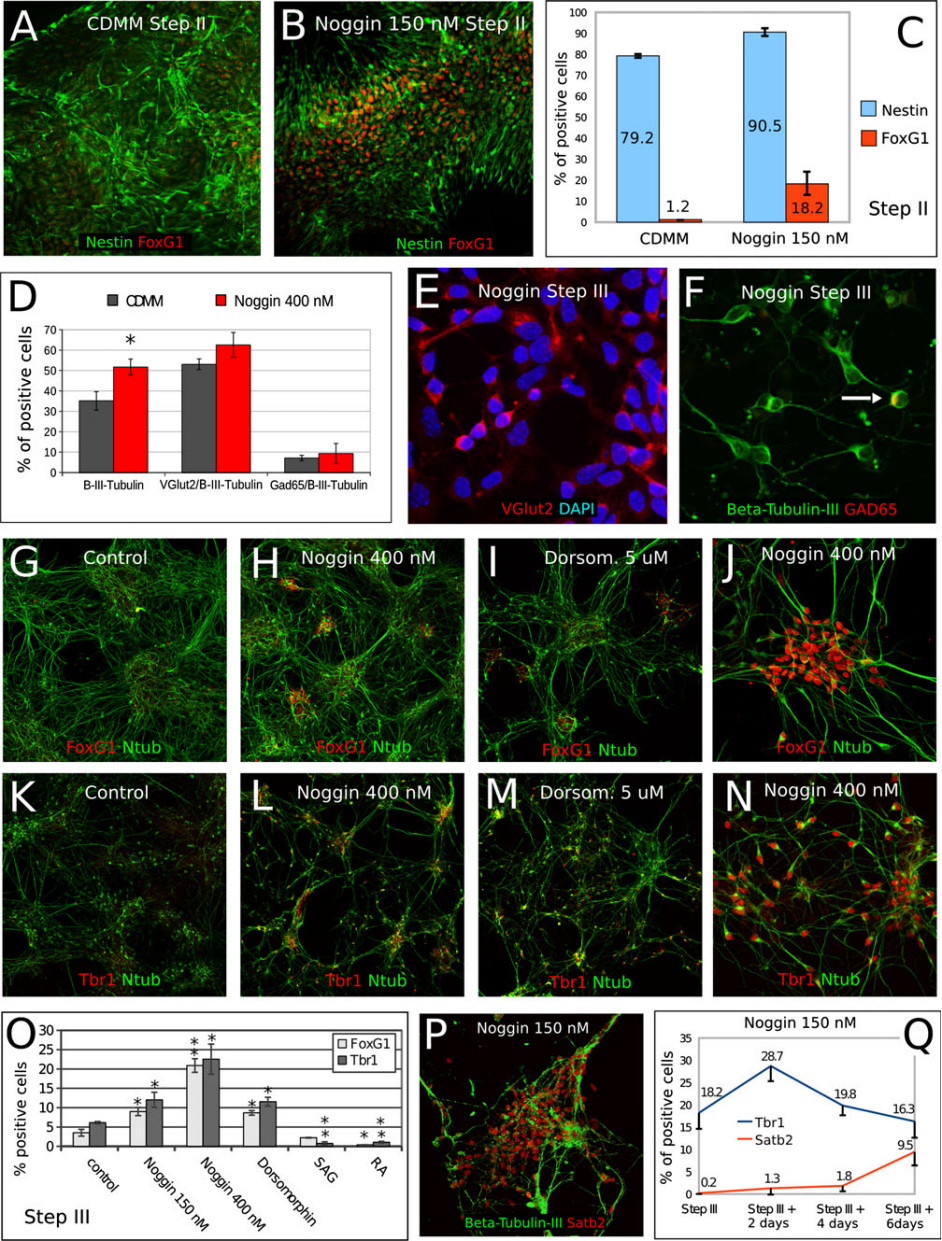
Effects of BMP inhibition on ESCs neural conversion and cell fate acquisition: **a–c** double immunocytodetection of Nestin (*green*) and FoxG1 (*red*) at the end of step II in ESCs cultured in CDMM (**a**) or in CDMM + Noggin (150 nM, **b**). FoxG1-positive cells were always co-labeled by Nestin. Numbers in (**c**) show ratios of Nestin-positive cells among total cells (*light blue bars*), or ratios of FoxG1-positive cells among Nestin-positive cells (*red* *bars*). **d–f** VGlut2 (*red* in **e**), β-III-Tubulin (*green* in **f**), and Gad65 (*red* in **f**) immunocytodetection and cell counts in Noggin-treated ESCs at step III + 4 days. *Arrow* in (**f**) indicates a β-III-Tubulin/Gad65 double positive cell. **d** The ratios of cells positive for the markers in (**e** and **f**). **g–o** Immunocytodetection of FoxG1 (*red* in **g–j**), Tbr1 (*red* in **k–n**) and acetylated-Tubulin (*green* in **g–n**) in ESCs cells at the end of step III after differentiation in CDMM (control; **g**, **k**), CDMM plus Noggin (400 nM; **h**, **l**, **j**, **n**) and Dorsomorphin (5 uM; **i**, **m**). **o** Cell ratios of FoxG1-positive and Tbr1-positive cells from culture conditions as in (**g–n**), and for ESCs treated with SAG, RA or 150 nM Noggin (not shown). **p** A group of neurons almost all positive for Satb2 nuclear staining. **q** Numbers show the ratios of Tbr1 or Satb2 positive cells over time in 150 nM Noggin-treated ESC cultures. *Error bars* standard error; *p < 0.05, **p < 0.01 (two-tailed Student’s *t* test)

We further investigated the nature of cells generated by Noggin-treated ESCs. At the end of step II, we found 79.2 % Nestin-positive neural progenitors in CDMM-differentiating ESCs, while Noggin treatment (150 nM) increased this ratio to 90.5 %. Notably, of the Nestin-positive progenitors in CDMM cultures only 1.2 % were positive for FoxG1, while Noggin treatment (150 nM) increased this ratio to 18.2 % (Fig. 6a–c). This implies that the majority of ESCs in CDMM become neural progenitors also without Noggin, but Noggin is necessary to acquire a telencephalic identity (see “Discussion”).

The ratio of β-III-Tubulin positive neurons in cultures treated with Noggin (400 nM) was slightly higher than the ratio in control cultures at the end of Step III (Fig. 6d). In both conditions, the majority of cells negative for β-III-Tubulin staining were Nestin-positive progenitors (not shown). Both control and Noggin-treated ESCs generated high ratios of VGlut2-positive glutamatergic neurons (Fig. 6d, e), whereas the ratios of GAD65-positive GABAergic neurons either in control or in Noggin-treated ESCs were lower (Fig. 6d, f). Noggin induced the expression of a number of genes coding for the isoforms of receptors for many different neurotransmitters, including GABA (Supplemental Table 6).

To investigate in detail the nature of cells generated by Noggin-treated ESCs, at the end of Step III, we analyzed at the cellular level the expression of FoxG1, which labels telencephalic neuronal progenitors [54], and Tbr1, the expression of which specifically identifies a sub-set of cortical neurons (Cajal-Retzius cells, subplate cells and glutamatergic neurons of the deep layers of the cerebral cortex; [29]). We compared the expression of the two proteins in control cells and in cells differentiated in the presence of Noggin (150 and 400 nM), Dorsomorphin (5 μM), SAG (100 nM) or of RA (10 μM) during step II. We found that, compared to control (Fig. 6g, k), ESCs treated at Step II with Noggin produced a higher ratio of both FoxG1-positive (Fig. 6h, j, o) and Tbr1-positive cells at step III (Fig. 6l, n, o). Consistently, the expression of both proteins was induced by Dorsomorphin and repressed by RA (Fig. 6i, m, o). Ventralization induced by SAG inhibited Tbr1 expression and left Foxg1 expression almost unchanged (Fig. 6o).

Notably, Noggin-induced expression of Tbr1, which marks earlier cortical neurons, was followed by the activation of Satb2 (Fig. 6p), which labels late-generated cortical neurons of layers 2/3. This suggests that Noggin-treated ESCs follow a differentiation schedule similar to that of in vivo cortical neurons (Fig. 6q).

We considered the possibility that Noggin acts by selecting cells committed to a cortical identity, which might be already present in ESC cultures maintained in serum + LIF. We thus assayed the effect of Noggin on ESCs selected in the absence of signals that might influence their differentiation potential. ESCs in which mitogen-activated protein kinase signaling and glycogen synthase kinase-3 (GSK3) are double-inhibited are homogeneous and pluripotent when cultured in a medium containing LIF but devoid of serum (2i ESCs; [57, 71]). In our protocol, 2i ESCs neuralization was slightly faster than the neuralization of ESC maintained in serum + LIF (Supplemental Figure SF7A, B). However, the expression of A/P and D/V markers in neural cells obtained by 2i ESCs was comparable to the expression in neural cells obtained by ESCs cultured in serum (Supplemental Figure SF7C–J). For this reason, we can exclude that our results might be influenced by some heterogeneity of the starting ESC population due to culture in serum-containing medium.

We characterized the identity of Noggin-treated ESCs in more detail by comparing their global gene expression profiles to the profiles of ESCs differentiated in other culture conditions, or to the profiles of embryonic brain regions. To this purpose, we performed microarray hybridization (see “Materials and methods”).

**Fig. 7:**
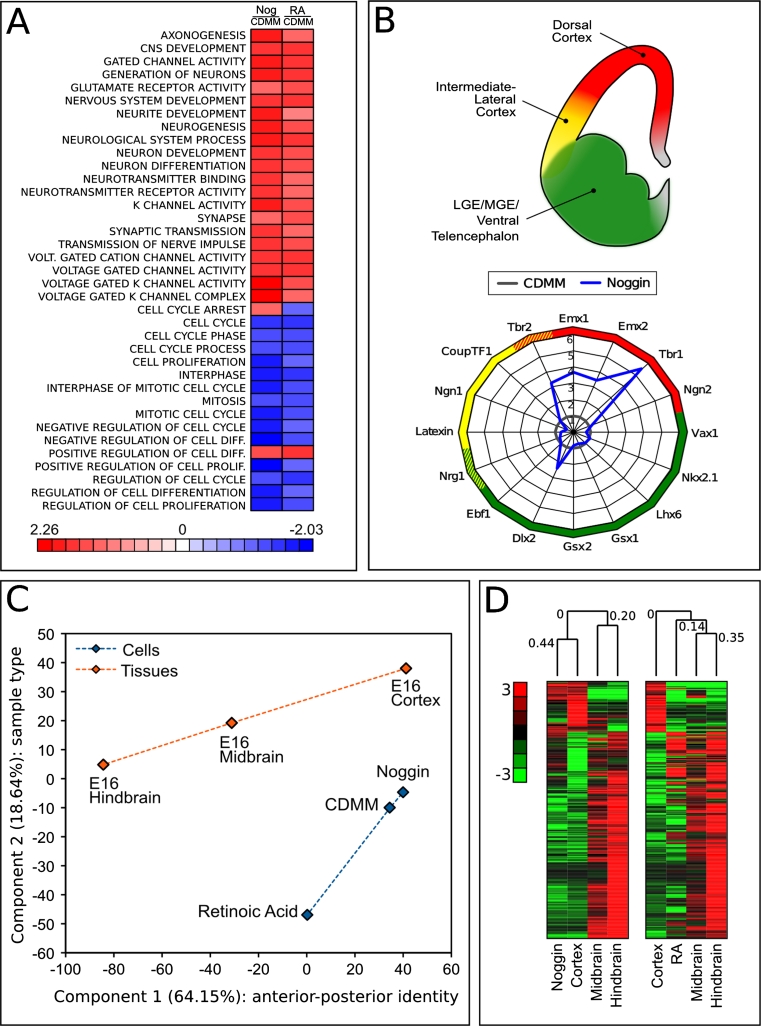
Gene expression profiling of differentiated ESCs: **a** Gene Set Enrichment Analysis of 400 nM Noggin-treated versus control ESCs (Nog/CDMM, first column) and 10 μM RA-treated versus control ESCs (RA/CDMM, second column), filtered for neuronal function/differentiation and control of cell cycle. Heat map color scale indicates gene set enrichment scores. **b** Gene expression fold change of selected forebrain markers (see Supplemental Table 7 for references) at step III in Noggin-treated ESCs compared to control (CDMM). **c** Principal component analysis of ESCs and E16 brain regions (see text for details). **d** Hierarchical gene clustering analysis of ESCs and E16 brain regions. The first 390 genes are shown (complete clustering is displayed in Supplemental Figure SF7B–D). Numbers over the branching report Euclidean distance correlation. Heat map color scale indicates normalized gene expression

As RA is a potent inducer of neuronal differentiation [24], we compared its action to that of Noggin on ESCs differentiation. We analyzed gene expression profiles using Gene Set Enrichment Analysis (GSEA). GSEA is a computational method which allows to identify, within predefined groups of genes (gene sets associated with particular cellular functions) whether a significant enrichment of regulated genes occurs when comparing two conditions [61]. Figure 7a shows gene ontology categories implicated in neuronal function/differentiation and cell cycle control. The color heat map displays gene set enrichment scores for Noggin-treated (400 nM) versus control ESCs (first column) and for RA-treated (10 uM) versus control ESCs (second column). Comparing Noggin to RA reveals that both molecules induce highly concordant effects, as seen by the upregulation of gene sets associated to neuronal differentiation and by the repression of gene sets related to cell proliferation and cell cycle progression.

We then investigated the effect of Noggin on positional identity. Noggin induced the expression of a number of dorsal–telencephalic markers and left almost unchanged the expression of intermediate-lateral or ventro-basal markers. This was evaluated by comparing the mRNA expression profile of ESCs treated with Noggin (400 nM) to that of control (CDMM-differentiating ESCs; Fig. 7b).

To study the effects of Noggin and RA on anterior/posterior (A/P) identity of ESCs, we selected a predefined subset of developmental genes known to pattern the A/P axis of the CNS, and we analyzed their expression, using RA treatment as a control for posteriorization of ESCs. A number of these genes were coherently regulated in Noggin-treated ESCs and E16 cortex, or in RA-treated ESCs and E16 hindbrain, suggesting a certain similarity of treated ESCs and corresponding brain regions (Supplemental Figure SF8A).

To further characterize ESCs positional identity, we extracted a list of genes that are differentially expressed along the A/P axis of developing brain. We chose 592 genes that were differentially expressed between E16 cortex and E16 hindbrain with absolute fold-change greater than, or equal to, 10-fold. This gene set (Supplemental Table 5) was first analyzed by principal component analysis (PCA) to assay the effect of Noggin on ESC positional identity. PCA is an unbiased method of analysis that projects data variability on a reduced number of orthogonal axes, such that the first axis captures the highest degree of variance in gene expression (Component 1), and subsequent axes (Component 2…n) correspond to successively decreasing variance. The components capturing the highest degrees of variance identify the qualities mostly discriminating among data populations.

Figure 7c shows a plot of the first two principal components, which account for 64.15 % (component 1) and for 18.64 % (component 2) of variance between samples. Component 2 discriminates ESCs (cyan items) from brain tissues (orange items). As expected by the nature of gene set selection, component 1 discriminates the A/P identity (dashed lines in Fig. 7c). Notably, Noggin-treated ESCs have more positive values on component 1 than control ESCs, confirming the anteriorizing effect of BMP inhibition. As an internal control of the analysis, RA-treated ESCs show an opposite trend, consistently with RA posteriorizing effect.

The same gene selection of 592 genes was used for hierarchical gene clustering analysis (see “Materials and Methods”) of either Noggin-treated or RA-treated ESCs, with the three brain regions (Fig. 7d; Supplemental Figure SF8B–D). We found that the gene expression profile of Noggin-treated ESCs clustered with that of cerebral cortex (0.44 correlation factor), whereas the RA profile clustered with midbrain/hindbrain profile, although with a lower extent (0.14 correlation factor). Regions of high concordance between the differentiated ESCs and the corresponding brain region are shown in Supplemental Figure SF8B–D and correspond to known genes of A/P patterning, including those genes whose expression was analyzed by RT-PCR.

We concluded that Noggin, in addition to its known role as neural inducer, plays a major role in establishing an anterior, cortical fate.

## Discussion

We have addressed the direct role of BMPs in anterior–posterior neural patterning. A role for BMP in inhibiting an anterior identity was suggested by many observations. Classical studies in lower vertebrates showed that BMP antagonism on *Xenopus* animal caps generates anterior neural structures [26, 38, 56]. In mouse, specific forebrain defects in mice mutant for BMP antagonists were shown [3]. However, this is to our knowledge the first study that directly addresses this issue in a systematic way in neuralized ESCs. We have established an original method of ESCs neuralization that permits to obtain fully differentiated neurons in a short time through the use of a chemically defined, minimal medium. These cells respond to RA and Shh by activating posterior and ventral pathways of differentiation, respectively. This is a strong evidence that in vitro they follow and respond the same signals found during in vivo embryonic development. We assayed the effect of BMP endogenously produced by neuralized ESCs on their own positional identity. The use of this in vitro differentiation method has allowed us to convincingly show that BMP signaling can influence the anterior–posterior neural patterning independently of signals from other germ-layers. In fact, neuralized ESCs spontaneously acquire a dorsal–telencephalic identity when deprived of endogenous BMPs. An important significance of our finding in the stem cells field consists in the possibility to obtain in vitro cortical neurons from pluripotent ESCs very rapidly and easily, without the need of any external signaling.

We found that ESCs cultured as adherent cells in a minimal medium without any added exogenous factors (CDMM), differentiated as neurons more efficiently than ESCs cultured in serum-containing medium (SCM) during the early phase of differentiation (step I). This is consistent with similar observations reported in the literature ([15, 20, 22, 65, 66]) and confirms the notion that a default program of neuronal differentiation of ESCs exists and can be inhibited by factors contained in serum. We do not know to what extent BMPs, which are present in serum [37], may account for its inhibitory effects.

Neurons generated by ESCs in CDMM express mid-brain markers, but we cannot exclude that a portion of them acquired a diencephalic identity. In fact, these neurons express Otx2 and Irx3, which are also expressed in caudal regions of the developing diencephalon. Moreover, BMP antagonists nearly completely repressed En1 but not Otx2 and Irx3, suggesting that some degree of diencephalic specification may be retained even following BMP inhibition. In any instances, an accurate comparison of their global gene expression profile to the global gene expression profile of dissected embryonic diencephalon is necessary to definitely address this point.

Noggin inhibited the action of endogenously produced, secreted BMPs and its action was specific, as confirmed by control experiments using BMPR1A-Fc and Dorsomorphin, which specifically block BMP pathway.

Noggin acted at two distinct levels of ESCs differentiation: it strengthened their spontaneous neural differentiation in a minimal medium and induced a telencephalic identity. Zfp521 (see “Introduction”) expression was highest in Noggin-treated cultures compared to any other culture conditions (Fig. 4b), confirming the crucial role of Noggin in ESCs neural conversion. However, Noggin induced only a slight increase of neural progenitor ratio compared to control, while supporting a dramatic increase of cells expressing the telencephalic marker FoxG1 (Fig. 6). This indicates that: (1) the removal of serum from our culture is per se sufficient to induce a high degree of neuralization, (2) although significant, the small increase in neural progenitors induced by high doses of Noggin cannot explain the dramatic increase of telencephalic cells, and (3) these results suggest a novel mechanism, whose molecular nature is still unknown, by which BMPs endogenously produced by differentiating ESCs directly act on the positional identity of the neural progenitors they spontaneously generate in a minimal medium.

Notably, we have induced comparable cortical commitment in ESCs which were propagated in chemically-defined conditions in the absence of serum (2i ESCs; [57, 71]) before using them for differentiation assays. Thus, the effect of Noggin on the positional identity of ESC-generated neurons is not due to the selection of cells committed to a cortical identity, which might be already present in ESC cultures maintained in serum + LIF.

 We speculate that the induction of the telencephalic transcription factors FoxG1 and Emx2 is sufficient to inhibit the expression of more posterior patterning genes as En1 and Otx2 through intrinsic molecular mechanisms, but the nature of such mechanisms has yet to be investigated.

To induce a cortical fate, some procedures make use of a feeder layer of stromal cells [32], or cell aggregation [15]. In these studies, the factors that were endogenously produced by cells in culture and that might have influenced ESCs differentiation were not identified. In one of these studies, ESCs cultured in a minimal medium at a very low density generated cells with morphological, electrophysiological, and molecular features of anterior neurons. These could be directed toward a cortical fate by treatment with the SHH antagonist Cyclopamine, although neither SHH secretion nor autocrine action of SHH were directly investigated [19]. We did not observe any effect of Cyclopamine on ESCs dorsoventral fate. However, we can confirm that ESCs can activate SHH signaling, as shown by the ventralizing effect we describe when adding a SHH agonist during step II. We hypothesize that, under the low density culture conditions employed by Gaspard et al. [19], an endogenous production of SHH that was not present in our culture condition was induced. In any case, ESCs differentiating as a monolayer of adherent cells in a minimal medium devoid of external signals were never able, to our knowledge, to induce a genuine cortical gene expression profile, as we on the contrary observed in our Noggin-treated cells.

The analysis of multiple markers is required to correctly determine CNS regional identity and exclude possible alternative fates in ESC-derived neural precursor cells [25]. To this purpose, we carried out a large-scale gene expression analysis of differentiated ESCs, using principal component analysis (PCA) and hierarchical clustering. Our main finding is that Noggin has a profound effect on the positional identity of ESCs-generated neurons, as it up-regulated the global gene expression of cortical genes and down-regulated that of midbrain and hindbrain genes. Thus, we reasoned that a telencephalic, possibly cortical, fate might be the default, intrinsic differentiation program of pluripotent cells when endogenous BMP signaling is inhibited. This finding reinforces the evidence obtained by the immunocytodetection of cortical cells markers such as Tbr1 and Satb2 (cortical neurons of deep and upper layers, respectively).

The molecular and embryological bases of neural tissue induction and brain patterning are beginning to emerge, indicating BMPs as key linking molecules [41, 63]. In our experimental model, endogenous BMPs were able to inhibit the expression of telencephalic genes, while at the same time allowing ESCs neuronal differentiation and high levels of expression of more posterior markers such as En1 and Otx2. We speculate that BMP activity, which is finely tuned in mouse developing prosencephalon [17], might regulate regional differences in embryonic fore-midbrain as well as it does in ESCs differentiating in a culture dish.

A revisited analysis of mammalian neural induction points to a model in which neural inducing signals called “activators” are proposed to impart both neural and anterior identity to the ectoderm. In this view, events that posteriorize the anterior neural tissue to generate the full range of CNS structures would occur later, by “tranformer” molecules [41, 67]. According to such a classical model, we speculate that a primitive neuronal-telencephalic fate of ESCs might be further transformed in midbrain or hindbrain fate by a secondary signaling of BMP or RA “transformers”, respectively.

Inhibition of BMP signaling appears to be a crucial step in forebrain induction, as shown by the severe defects in the development of the prosencephalon of mice double mutants of the BMP inhibitors chordin and noggin [3]. However, dual inhibition of Wnt and BMP signals has been proposed to be necessary to confer head organizer activity both in zebrafish [31], *Xenopus* [21, 42] and mouse [14]. Although we observed a robust activation of cortical markers and a strong repression of midbrain genes with the sole inhibition of BMP, we cannot exclude that differentiating ESCs produce and are sensitive to Wnts and that Wnt inhibition might be synergistic with BMP inhibition in inducing a cortical fate in ESCs. Endogenous Wnt activity might explain why the ratios of ESCs treated with high doses of Noggin that express markers of cortical progenitors (FoxG1, 22 %) and of cortical neurons (Tbr1 and Satb2, 30 % in total) at the end of step III do not account for the total number of Sox1-positive cells neuralized at step II (90.5 %). Our model of ESCs neuralization might allow us to experimentally address this point and to dissect the role of other pathways involved in neural patterning better than other in vivo systems.

A crucial role for BMP in patterning neural structures has been recently suggested in vitro, as pluripotent cells of *Xenopus* animal caps acquired anterior neural fate when treated with high doses of Noggin [39, 64]. In fact, our results are consistent with this observation and point to the existence of a default, intrinsic program of differentiation of pluripotent cells that has been conserved through vertebrate evolution and is both neuronal and anterior.

## Electronic supplementary material

Below is the link to the electronic supplementary material.

Supplementary material 1 (PDF 3205 kb)

Supplementary material 2 (XLS 11 kb)

Supplementary material 3 (XLS 112 kb)

Supplementary material 4 (XLS 554 kb)

Supplementary material 5 (XLS 61 kb)

Supplementary material 6 (XLS 140 kb)

Supplementary material 7 (XLS 12 kb)

Supplementary material 8 (XLS 11 kb)
