# Supplementary material for: The positional identity of mouse ES cell-generated neurons is affected by BMP signaling
Source: Cell Mol Life Sci. 2012 Oct 16;70(6):1095–111. doi: 10.1007/s00018-012-1182-3 (PMC3578729; doi:10.1007/s00018-012-1182-3)
Supplement: Supplementary file 1 — Supplementary material 1 (PDF 3205 kb) [file 18_2012_1182_MOESM1_ESM.pdf]

Bertacchi et al., 2012 - Supplemental Figures

SF1 BMP2 ELISA calibration

Standard curve showing linear correlation between BMP2 concentration and absorbance at 450 nm, after colorimetric solid phase ELISA quantification in the range 0-1000 pg/ml (Pearson correlation coefficient of regression R^2=0.999).

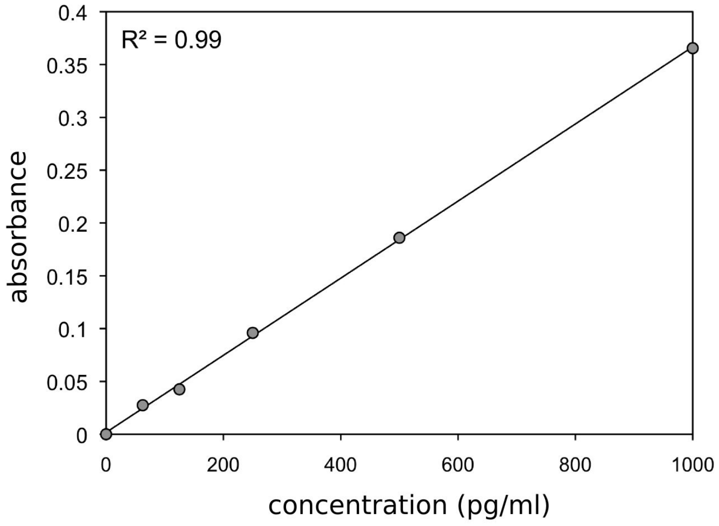

Supplemental Figure 1 - Cremisi

SF2 Characterization of ESCs differentiation

A-G show Beta-Tubulin-III and Nestin immunodetection identifying neurons and neural progenitor cells, respectively, in ESCs aggregated (step I) in CDMM (A,C,E,F) or SCM (B,D,G) and then differentiated in CDMM. Cells were analyzed at the end of step II (A,B), after 5 days of culture as aggregates (C,D), or at the end of step III (E-G). H shows mRNA fold changes of progenitor cell markers (Nestin) and neuronal markers (Beta-Tubulin-III, Ncam) as evaluated by RT-PCR (normalized on expanding ESCs, undifferentiated) at the end of step III in different culture conditions as in E-G. Error bars: standard error. Asterisks: * $p < 0.05$, ** $p < 0.01$, *** $p < 0.001$ (Randomization test, REST software). I, immunocytodetection for Beta-Tubulin-III-positive neurons (green). J-M Beta-Tubulin-III marked multipolar, pyramidal-like, bipolar and unipolar neurons, respectively. N,O, Beta-Tubulin-III-positive neurons (red immunocytodetection) showing alpha-Internexin-positive (green) varicosities (white arrows; N) or punctate Synaptophysin staining (green; O).

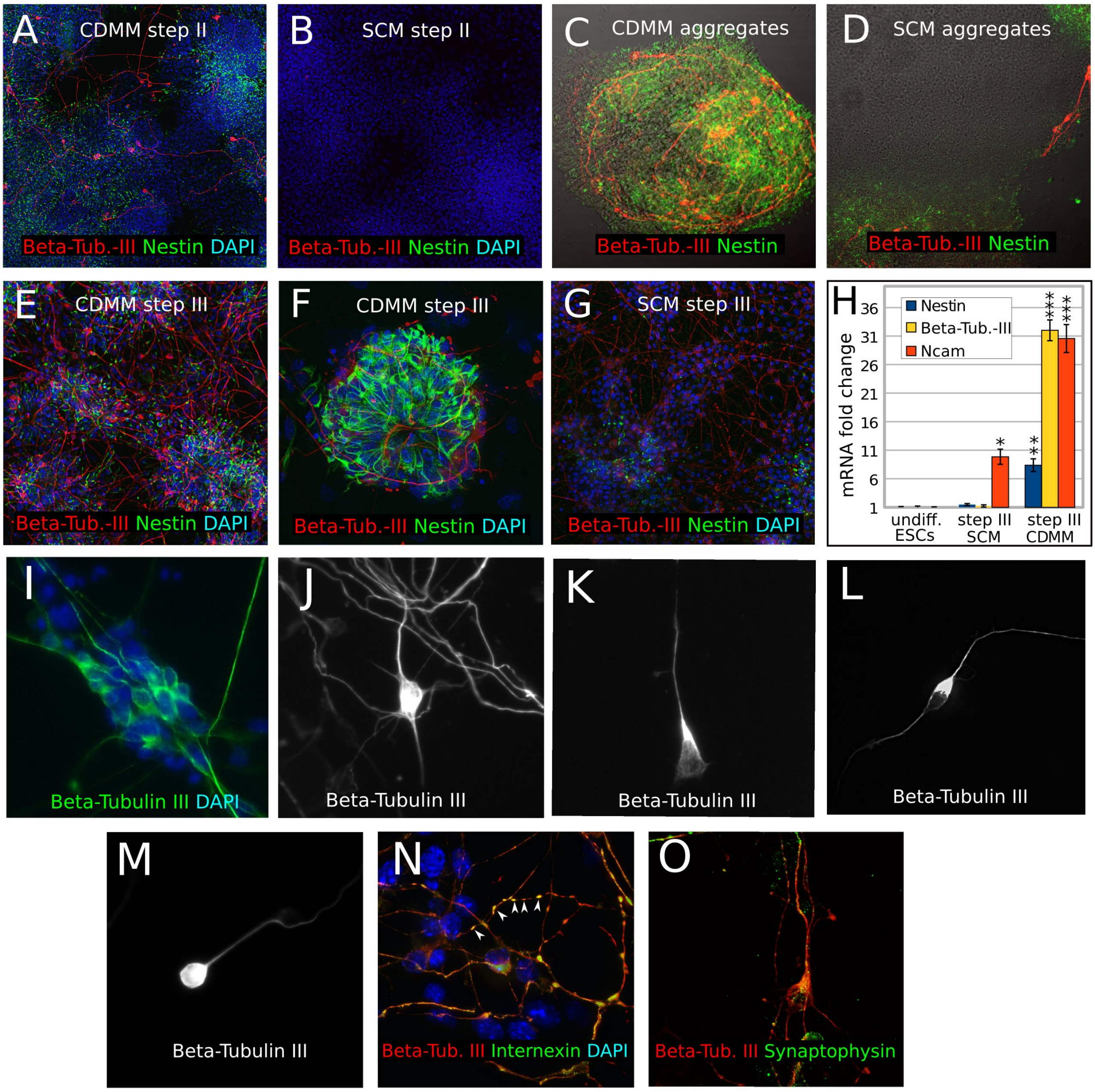

Supplemental Figure 2 - Cremisi

SF3 BMP signaling in differentiating ESCs

A-C, RT-PCR analysis. A shows mRNA relative expression of BMP receptors 1A and 1B in ESCs at different steps of differentiation, MSC and RAW cells (ratio over Beta-actin). B shows the effects of Noggin and Dorsomorphin on ID1 mRNA expression (normalized on CDMM). C, bars indicate mRNA relative expression of neural marker (Nestin) and pan-neuronal markers (NFL, Beta-Tubulin-III, Ncam, Pax6) in undifferentiated ESCs, in ESCs at step III after differentiation in CDMM, or after treatment throughout step II with 50 ng/ml BMP4 (normalized on expanding ESCs). Error bars: standard error.

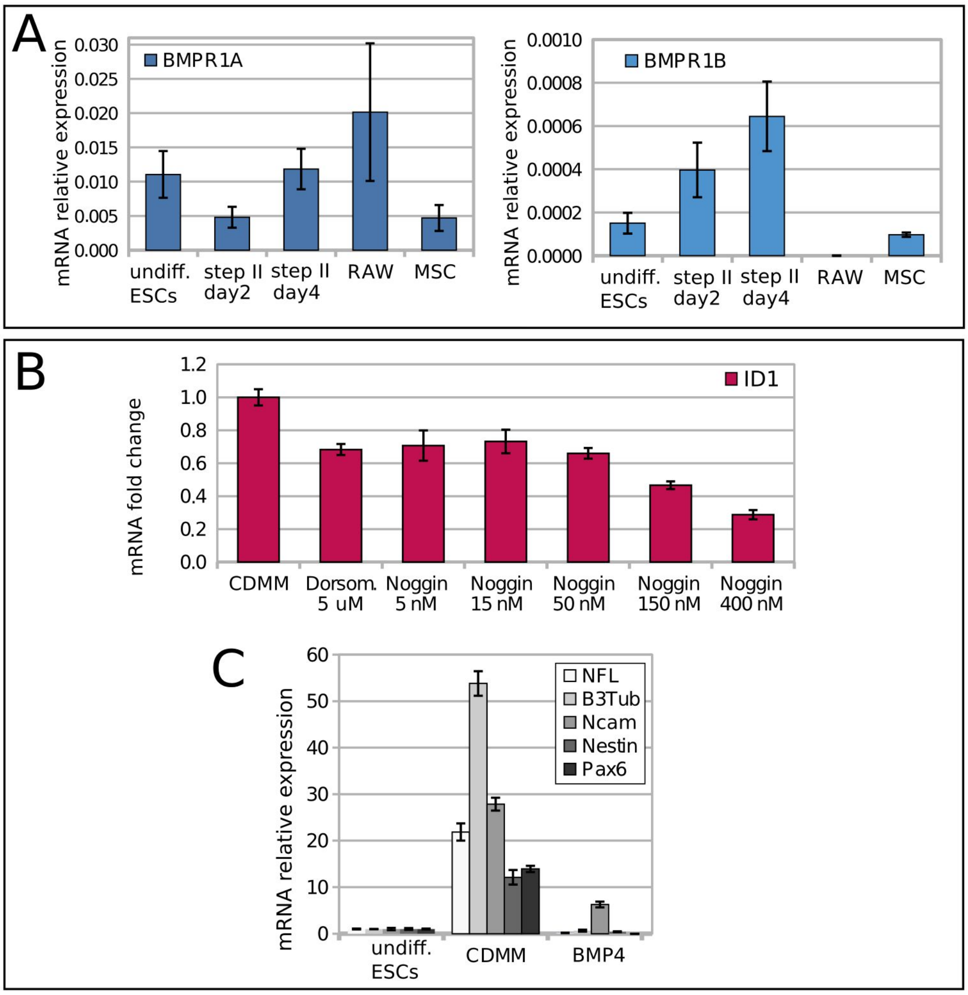

Supplemental Figure 3 - Cremisi

SF4 A/P and D/V characterization of differentiating ESCs

A, A/P (color code) patterning of mouse embryonic brain, identifying the regions of gene expression. Fb: forebrain, Mb: midbrain, Hb: hindbrain, SC: spinal cord. Values indicate mRNA expression fold changes in ESCs at the end of step III after exposure to different RA concentration throughout step II, as evaluated by RT-PCR (expression normalized on control, CDMM). B, V/D index, calculated as Nkx2.1/Pax6 mRNA ratio measured by RT-PCR at different time points and in different culture conditions or brain regions, as indicated (expression normalized on ventral forebrain level). The high variability of Nkx2.1/Pax6 mRNA ratio in undifferentiated ESCs is due to the very low levels of expression of Nkx2.1 and Pax6 mRNA in these cells. C shows RT-PCR analysis of Shh relative expression in ESCs at different times of differentiation and in different culture conditions, as indicated (ratio over Beta-Actin). Noggin, Noggin 400 nM; SAG, Shh agonist (100 nM); Cyc, Cyclopamine 10 uM. Error bars: standard error.

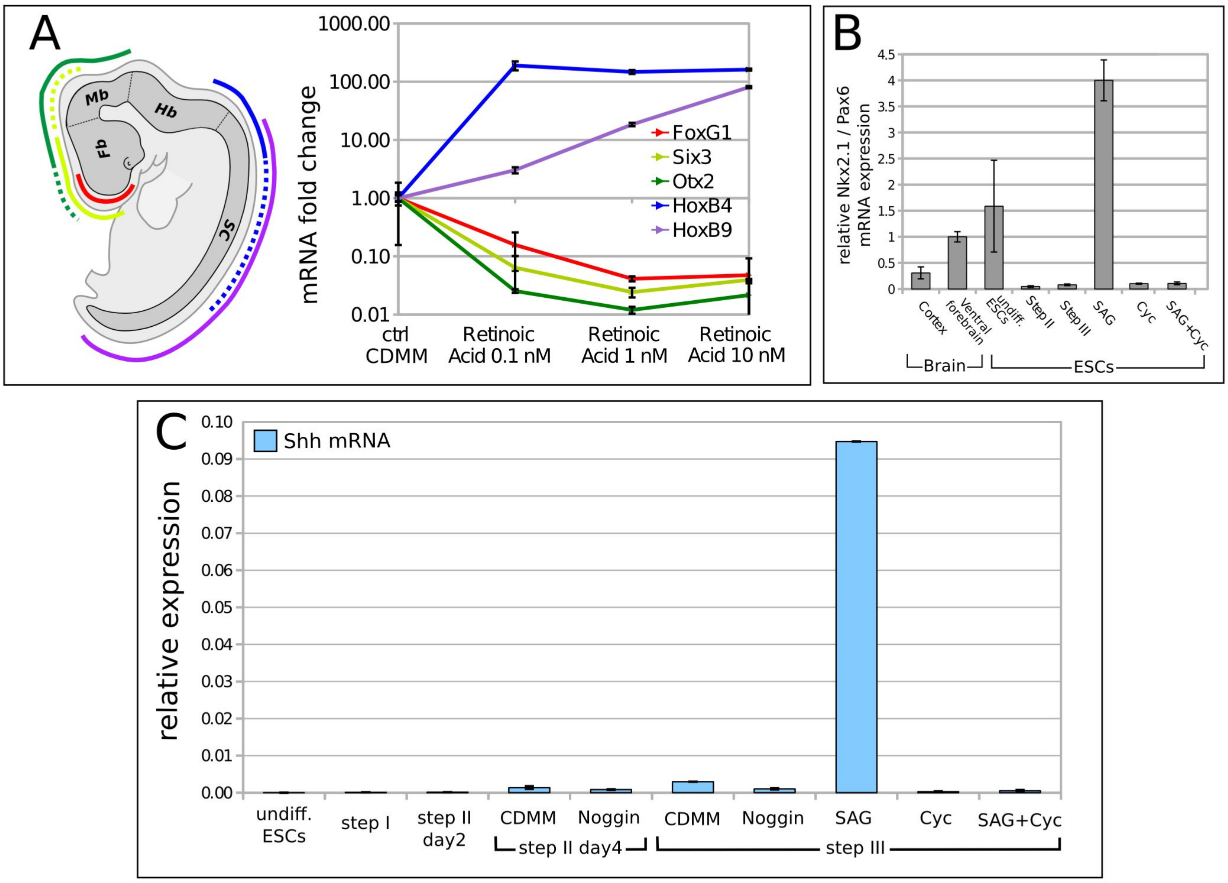

Supplemental Figure 4 - Cremisi

SF5 Temporal analysis of BMP effects in differentiating ESCs

A, Graph shows RT-PCR mRNA quantification of FoxG1, Otx2 and En1 in ESCs at early stage (end of step II) after differentiation in CDMM (0) or in CDMM plus 5 to 400 nM Noggin (normalized on maximum expression). B,C, RT-PCR mRNA quantification of forebrain/cortical markers (B, normalized on maximum expression) or hindbrain/spinal cord markers (C, ratio over Beta-Actin), in cells as in A. D, RT-PCR mRNA quantification of forebrain/cortical markers (blue colors) and mesencephalic markers (orange colors) in ESCs at the end of step III and at a late stage of differentiation (step III plus 4 days), after differentiation in CDMM (Control) or in CDMM plus 150 nM Noggin (normalized on maximum expression). Error bars: standard error.

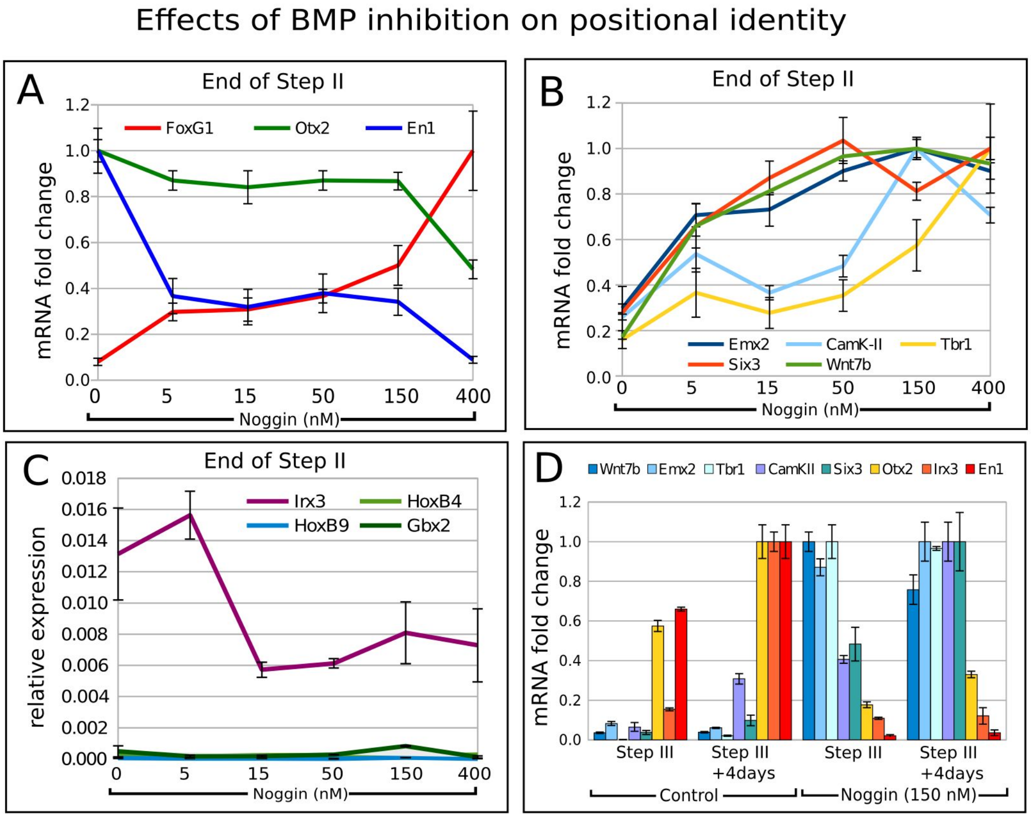

Supplemental Figure 5 - Cremisi

SF6 Dorsomorphin and BMPR1A-Fc mimic Noggin effects on ESCs differentiation

A, protein three-dimensional structures rendered with Pymol (http://www.pymol.org/). Molecular coordinates of Noggin-BMP7 and BMPR1A-BMP2 complexes, as submitted by Groppe et al., (2002) and Keller et al. (2004), were found in Protein Data Bank (http://www.pdb.org/). B, TGF-beta family pathways. Noggin, BMPR1A-Fc and chemical drugs affecting signal transduction are indicated. C shows the effects of BMPR1A-Fc treatment on ID1 mRNA expression, as evaluated by RT-PCR analysis (normalized on CDMM). D, flow-cytometry analysis of Sox1-GFP ESCs at day 2 of step II after culture in CDMM, or CDMM + 37.5 nM BMPR1A-Fc. Bars in E show mRNA fold change of A/P patterning genes in ESCs at step III after differentiation in different culture conditions (normalized on CDMM). Drugs were added throughout step II. F shows the effects of treatment during step II with BMPR1A-Fc on FoxG1, Otx2 and En1 expression, as evaluated by RT-PCR at the end of step III. Error bars: standard error.

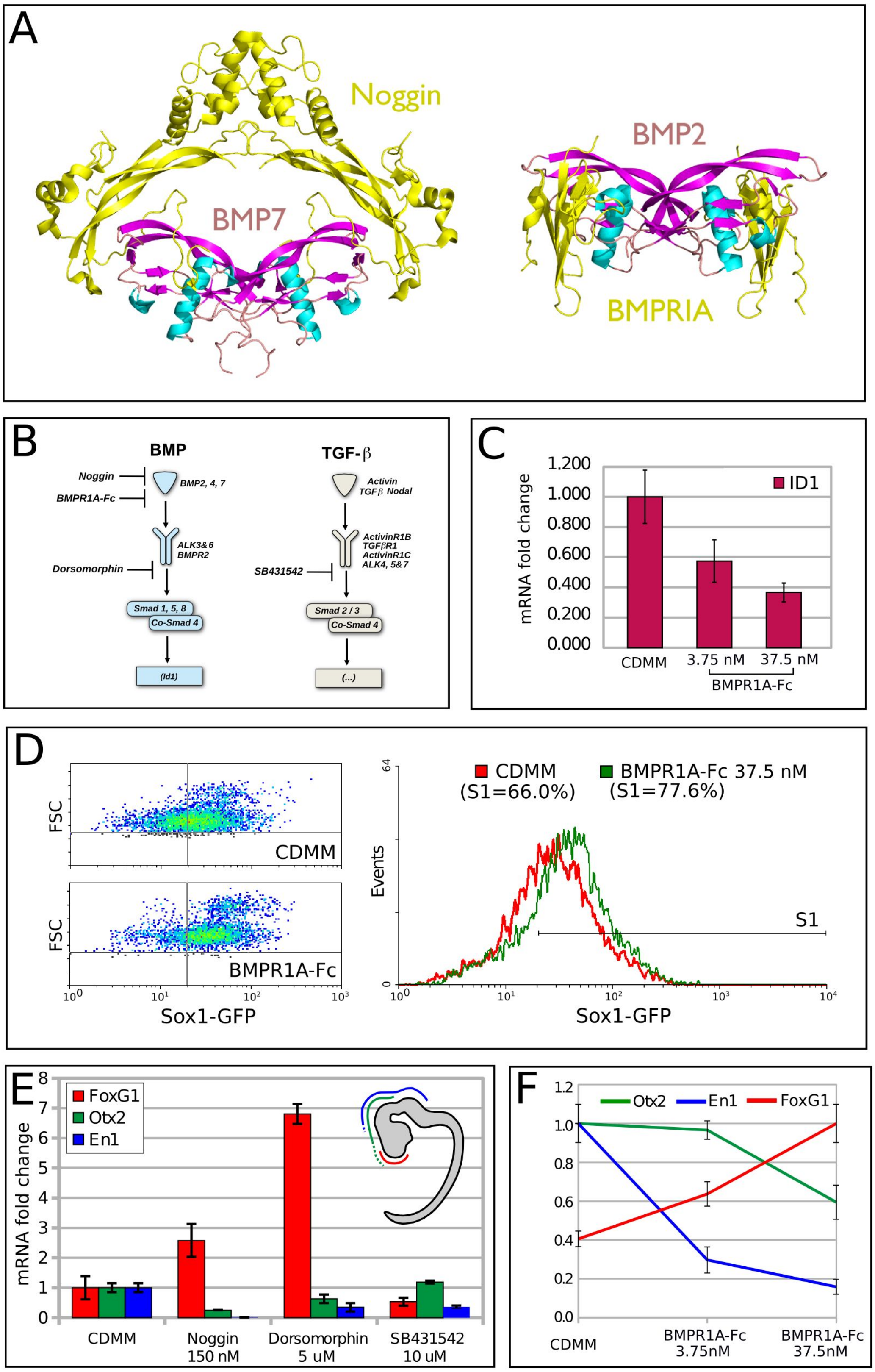

Supplemental Figure 6 - Cremisi

SF7 Neural induction and positional identity of 2i ESCs-derived neural cells

A, Histogram shows RT-PCR mRNA quantification of neuronal markers (Beta-Tubulin-III, Ncam and NFL) and progenitor cell marker (Nestin) during differentiation protocol, comparing the potential of ESCs previously cultured in serum+LIF (ESCs) to the potential of ESCs previously cultured in 2i+LIF (2i ESCs). Values are normalized on maximum expression. There is no significant difference in the expression of these markers between ESCs and 2i ESCs, except for the expression of Ncam, which was slightly lower at step III in 2i ESCs (64%) compared to ESCs.

B, Flow-cytometry analysis of Sox1::GFP ESCs at different time points of the neuralization protocol, as indicated. ESCs cultured in serum+LIF (ESCs, blue bars) are compared to ESCs cultured in 2i+LIF (2i ESCs, red bars). Sox1-driven GFP is almost not expressed in undifferentiated 2i ESCs and expressed at low levels in undifferentiated ESCs, it is activated earlier in 2i ESCs compared to ESCs by the differentiation protocol and it is expressed at similar levels at the end of step II. This is consistent with a higher homogeneity and a faster neuralization of 2iESCs compared to ESCs.

C-G, immunocytodetection of Pax6 (C,D) and Nkx2.1 (E,F) at the end of step II in 2i ESCs differentiated in CDMM (control, C and E) or in CDMM+SAG (D,F). Numbers in G show fractions of Pax6-positive cells (light grey bars) and Nkx2.1-positive cells (dark grey bars), in 2i ESCs differentiated in CDMM, CDMM+SAG and CDMM+Cyclopamine (Cyc). These results are consistent with the results obtained with ESCs (Figure 4C,D).

H shows FoxG1, En1 and Otx2 expression, as evaluated by RT-PCR at the end of Step II (normalized on maximum expression), after differentiation of 2i ESCs in CDMM (Control) or in CDMM plus 150 nM Noggin. I, histogram shows RT-PCR mRNA quantification of forebrain/cortical markers (blue colors) or more posterior marker Irx3 (orange color), in cells as in H at the end of step III. Values are normalized on maximum expression. J, Cell percentages of FoxG1-positive and Tbr1-positive cells at the end of step III, from culture conditions as in H and I. Error bars: standard error. These results are consistent with the results obtained with ESCs (Figure 5 and Figure 6O).

Methods: for 2i+LIF selection, ESCs (lines E14Tg2A, passages 32-39) and 46C (transgenic Sox1-GFP ESC kindly provided by A. Smith, University of Cambridge, UK, passages 33-40) were cultured as described in

Ying et al., 2008. Briefly, cells were selected for three passages (6 days of culture) in GMEM (Sigma) supplemented with 2mM Glutamine, 1mM sodium Pyruvate, 1mM non-essential amino acids, 0.05mM β-mercaptoethanol, 100 U/ml Penicillin/Streptomycin, 1000 U/ml recombinant mouse LIF (Invitrogen), N2/B27 (no vitamin A; Invitrogen) and 2i inhibitors (1uM PD0335901 and 3 uM CHIR99021, Santa Cruz). Control ESCs were maintained in parallel in serum-containing medium for the same number of passages. Oct4 and Nanog upregulation in 2i ESCs after selection was confirmed by immunocytodetection (not shown).

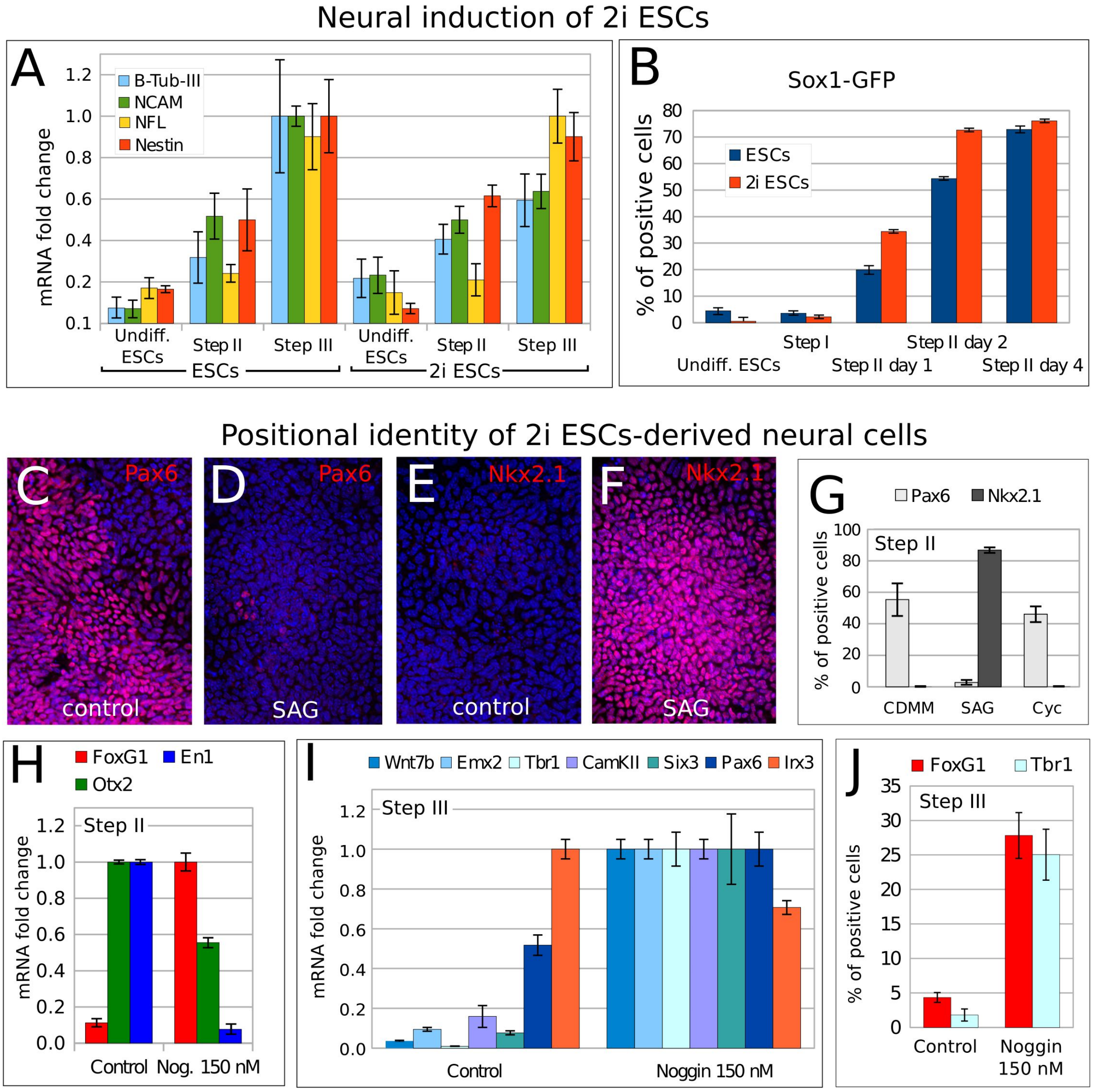

Supplemental Figure 7 - Cremisi

SF8 Gene expression clustering of ESCs and E16 brain tissues

A, expression profiling of a predefined subset of developmentally expressed genes known to pattern the A/P axis of the central nervous system (CNS). The figure shows a heat map of the expression analysis of such genes, which are differentially regulated in Noggin-treated cells compared to control (Nog/CDMM; see Supplemental Table 2) and in E16 cortex compared to E16 midbrain (Cortex/Mid.), or that are differentially regulated in RA-treated cells compared to control (RA/CDMM; see Supplemental Table 3) and in hindbrain compared to E16 midbrain (Hind/Mid.). Some of these genes were coherently regulated in Noggin treated ESCs and E16 cortex, or in RA treated ESCs and E16 hindbrain, suggesting a similar identity of treated ESCs and corresponding brain regions. B-D, Hierarchical gene clustering analysis of ESCs at step III, after differentiation in defined culture conditions, and E16 brain regions. Numbers over the branching report Euclidean distance correlation. Enlarged aspects indicated by brackets are regions of high concordance between differentiated ESCs and the corresponding brain region. Genes shown in these enlarged regions are mainly developmental genes whose expression patterns the CNS A/P axis and that significantly contributed to the clustering. Heat map color scale indicates normalized gene expression.

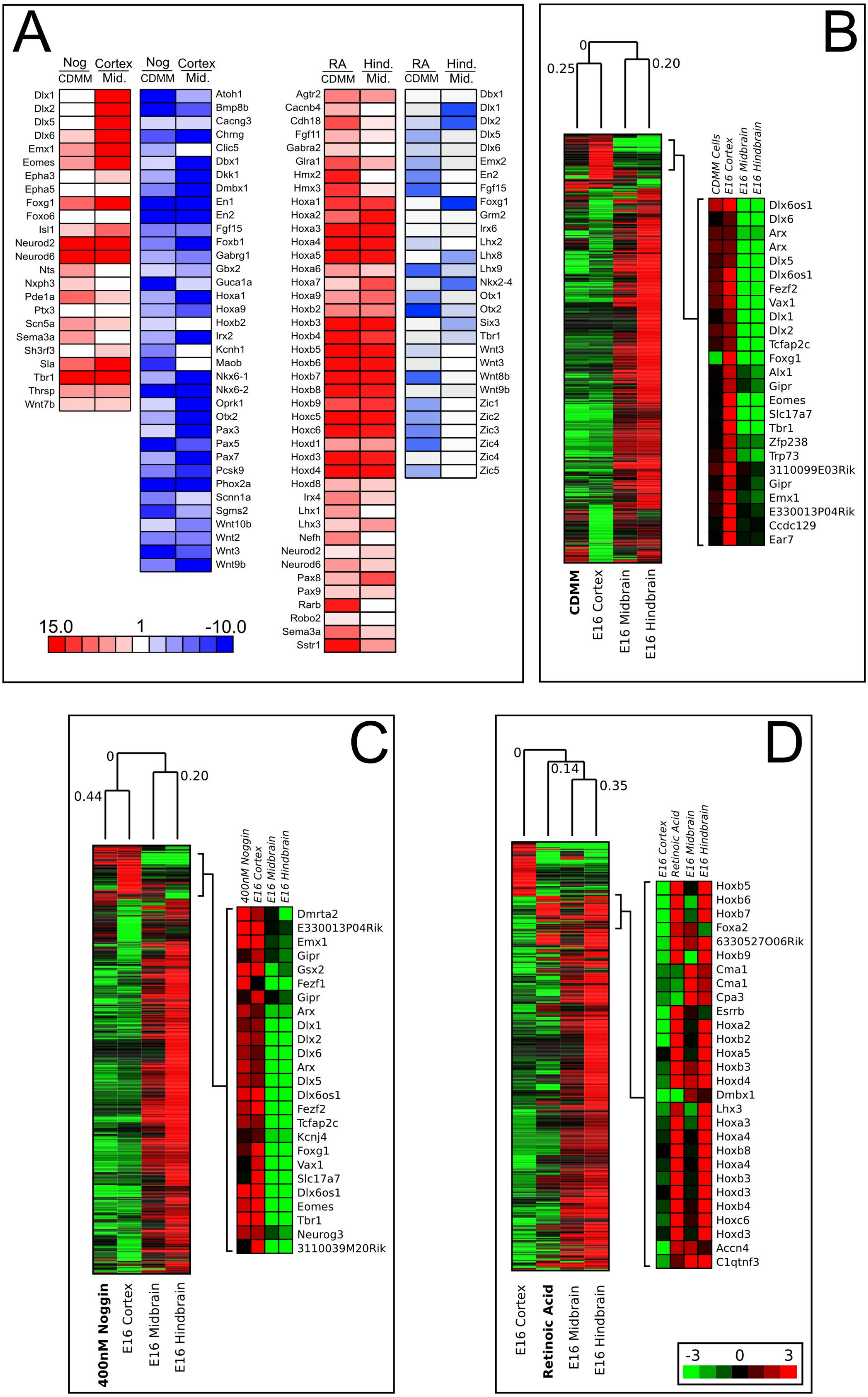

Supplemental Figure 8 - Cremisi
